# Medical expenditure for strabismus: a hospital-based retrospective survey

**DOI:** 10.1186/s12962-022-00363-2

**Published:** 2022-06-25

**Authors:** Lei Yang, Yiduo Min, Zhiyan Jia, Yupeng Wang, Rihui Zhang, Bitong Sun

**Affiliations:** 1 Department of Ophthalmology, The First Affiliated Hospital of Harbin Medical University, Harbin, Heilongjiang China; 2 Department of Biostatistics, Harbin Medical University, Harbin, Heilongjiang China

**Keywords:** Strabismus, Surgery, Medical expenditure, China

## Abstract

**Background and aims:**

The misconception of the purpose of strabismus treatment has, on the one hand, affected the motivation of strabismus patients to seek care and, on the other hand, has resulted in strabismus not being covered by health insurance, both of which interact to limit the motivation of strabismus patients and also impose a financial burden on strabismus patients. Previous studies on the cost of strabismus had only addressed the cost utility and functional and psychosocial benefits of strabismus surgery. The aim of this study was to estimate the direct medical expenditure incurred for strabismus surgery and analyze the trend for the period 2014–2019 using the data collected by local eye hospitals in northeast China.

**Methods:**

This study was based on 6-year strabismus medical expenditure data collected from the eye hospital of the first affiliated hospital of Harbin medical university, covering 3596 strabismus patients who had strabismus surgery. All medical expenditure data were adjusted to 2014 using China’s annual consumer price index to remove the effects of inflation.

**Results:**

The average direct medical expenditure for strabismus cares (in 2014) was 5309.6 CNY (US$870.4), and the annual growth rates from 2015 to 2019 (compared with the previous year) were 9.3, 7.7, 21.7, 14.5, and 4.3%, respectively. Surgical expenses accounted for the highest proportion (33.1%) of the total medical expenses followed by examinations expenses (19.7%) and medical consumables expenses (18.7%). The regression coefficient for general anaesthesia was 1804.5 and age was less than 0.

**Conclusion:**

The average direct medical expenditure for strabismus increases year by year, and the growth rate is rapid. Anesthesia was the most important factor increasing medical cost, and age was negatively correlated with cost.

## Background

Strabismus is a common eye disease, with a prevalence of 1–4% in the population [1–3]. Many factors affect the prevalence of strabismus, such as age, gender, region, refraction, etc. There are different views on the trend of strabismus prevalence with age. Kvarnström et al. [4] considered that age is an important factor of the prevalence of strabismus. However, some studies have shown that age does not affect the prevalence of strabismus [5, 6]. Refractive error is also one of the important factors influencing the prevalence of strabismus. The prevalence of strabismus in western countries is higher than that in Asian countries [7], probably because of the high incidence of hyperopia [8, 9]. In addition, the higher prevalence of strabismus in female may be related to the higher prevalence of hyperopia in female [10]. However, some studies suggest that there is no gender difference in the prevalence of strabismus [11, 12]. Gender differences may be due to other factors, such as age distribution, race, and genetic and environmental factors of the study population [7]. It has been reported that the prevalence of strabismus has no significant correlation with age, gender, place of residence and refractive error [13]. The risk factors of strabismus include premature birth, low birth weight, smoking during pregnancy, family history, etc. [12, 14, 15].

The effects of strabismus are multidimensional, with potentially detrimental effects on overall health and quality of life [1, 3, 16–20]. Even the quality of life of the family members of strabismus patients may decline [21, 22]. Most types of strabismus usually require surgical treatment, and studies have demonstrated the clinical and functional benefits of strabismus surgery [20, 23–26]. It can improve binocular vision and psychosocial functioning. Even in adults with long-standing strabismus, binocular vision improves after strabismus surgery, and stereopsis may be restored in some patients [24, 27]. However, strabismus surgery is often labeled as “cosmetic”, that is the reason why strabismus is not in health insurance coverage in China, and even many ophthalmologists hold the same misconception [28].This prevents strabismus patients from getting treatment in early stages.

Accurate estimates of strabismus treatment costs are necessary for health care. Information on hospitalization costs associated with strabismus surgery is equally important for clinicians to increase their understanding of the economic impact of surgical interventions, as well as for patients to get information of the accurate manner of expected medical costs. Previous studies have mostly paid attention to assess the cost utility and functional and psychosocial benefits of strabismus surgery [29–32], but the year-to-year trends in hospitalization costs for strabismus and the associated influencing factors have not been systematically studied. The purpose of this study was to present a 6 -year trend and influencing factor analysis of the overall hospitalization costs associated with strabismus surgery.

## Methods

### Data source and sample size

The data were obtained from the information system database of the eye hospital of the first affiliated hospital of Harbin medical university. The eye hospital is the best eye hospital in Northeast China. Information on the inpatient medical expenses of all strabismus patients from January 2014 to July 2019 was extracted from the database, with a total of 3819 strabismus patients.

### Medical expenditure data inclusion/exclusion criteria

The inclusion of medical expenditure data for hospitalized patients is subject to the following conditions simultaneously: (1) a diagnosis of strabismus requiring surgery; (2) a single diagnosis with no other medical treatment costs incurred; (3) the discharge date is From January 1st, 2014 to July 31st, 2019; (4) the patient’s basic information, cost information, and clinical information (clinical diagnosis, treatment plan, anesthesia, etc.) are complete.

Exclusion criteria: (1) cases with two or more diseases; (2) patients with incomplete information.

A total of 3819 inpatients with strabismus from 2014 to 2019 year were extracted from the database. 31 cases with incomplete information and 192 cases with non-single diagnosis were excluded, finally 3596 cases of strabismus being included in this study.

Categories of medical expenses for this study: surgical treatment (surgery, anaesthesia), drug, medical consumables (consumables for treatment, consumables for surgery), examinations (laboratory test, imaging and clinical diagnostic items), others (general medical service, general treatment operation, nursing and other).

### Data analysis

All costs are reported in China Yuan (CNY) based on the value in 2014, and are calculated based on the Consumer Price Index (CPI) for urban residents in Heilongjiang Province, with price adjustments for yearly medical expenses. Statistical analysis was performed using SAS 9.4 and SPSS 24.0 software. To analyze the changes of various expenses in different years: (1) to calculate the mean and standard deviation of costs for each year; (2) the cost was used as the dependent variable, the CPI as the covariate, and the year was included in the linear regression model as a continuous variable, and the P value of the linear trend test of the cost change with the year was obtained; (3) the cost was used as the dependent variable, the CPI as the covariate, and the year was included in the regression model as a categorical variable (2014 as the reference level), and covariance analysis was performed. Partial correlation was used to analyze the relationship between the total cost and sub-cost for each year, adjusting the CPI, and calculating the Spearman’s correlation coefficient. Nonparametric tests and univariate linear regression were used to analyze the relationship between total cost and gender, funding, age, length of hospital stay. On the basis of univariate analysis, multiple linear regression was used to analyze the influence of each factor on the total cost.

Values of P < 0.05 were considered statistically significant.

## Results

### Sample characteristics

A total of 3596 strabismus patients with strabismus were included for the period 2014–2019 (Table 1, Page 27).Male:female = 1.11:1. The youngest patient was 1 year old and the oldest was 71 years old. In terms of funding, the most of patients (83.4%) were fully self-funded. General anaesthesia was usually used in patients who cannot tolerate surgery, including extraocular muscle paralysis (2053 cases, 57.0%), and 1465 patients under 12 years old, accounting for 93.4%. The local anesthesia method in this study is the dropping of obucaine hydrochloride in conjunctival sac combined with subconjunctival injection of lidocaine. About the length of hospital stay, the lowest day was 2 days, the most day was 13 days, and the average day was 4.4 days.

**Table 1:** Characteristics of inpatient cases incorporating strabismus, 2014–2019

Variables	Results (n = 3596)
Gender	
Female	1704 (47.0%)
Male	1892 (53.0%)
Average age at surgery	17.9
Age of surgery	
≤ 12	1568 (48.4%)
13–24	1240 (34.5%)
25–60	767 (21.3%)
> 60	21 (0.6%)
Length of stay	
Median	4 days
Average	4.4 days
Funding	
Self-funded	3004 (83.4%)
Basic medical insurance for urban residents	149 (4.1%)
Urban employee basic medical insurance	32 (0.8%)
Other social insurance	128 (3.5%)
New rural cooperative medical insurance	124 (3.4%)
Other	159 (4.4%)
Anesthesia	
General anaesthesia	2053 (57.1%)
Local anaesthesia	1543 (42.9%)

### The changing trend of hospitalization expenses for strabismus patients

**Fig. 1:**
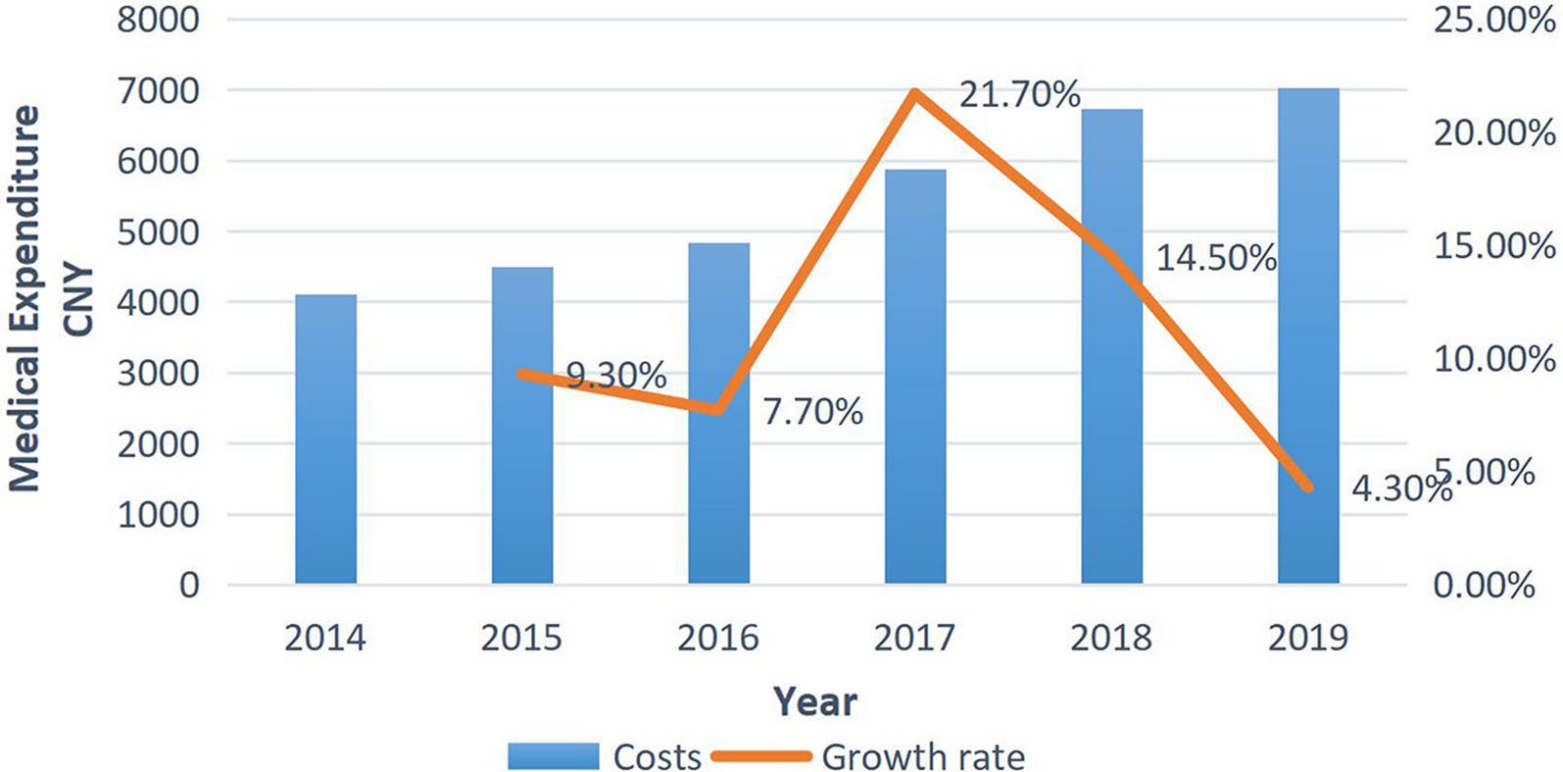
Trend in total hospital costs for strabismus patients

**Fig. 2:**
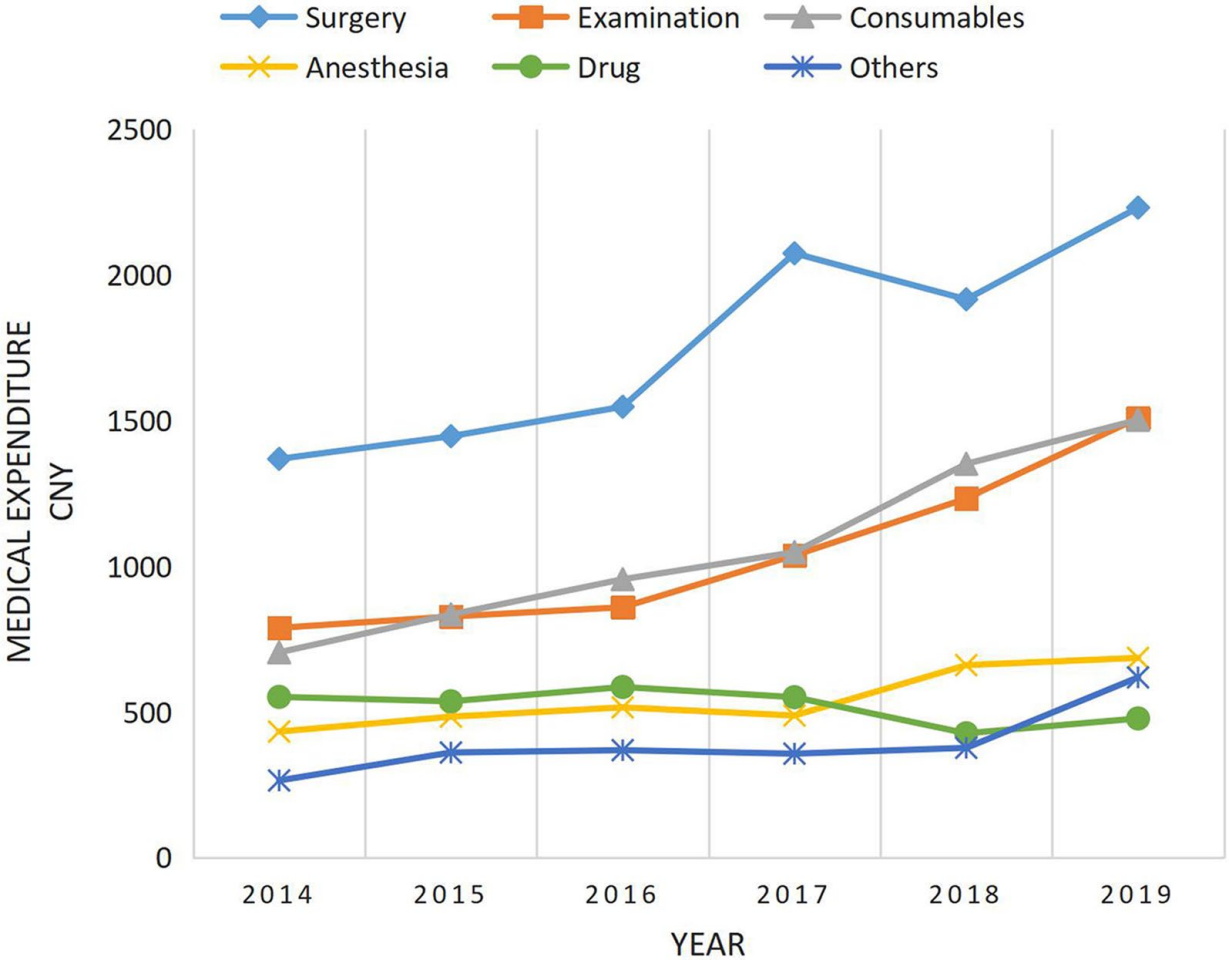
Trends of sub-costs of hospitalization expenses for strabismus patients, 2014–2019

The average cost of hospitalization was 5,309.6CNY (US$870.4) increasing year by year, with a faster rate of growth in 2017 and 2018 (Fig. 1). Expenditure percentages for drugs decreased over time, with the largest reductions in 2017(6.0%) and 2018 (22.5%). Medical consumables costs and examinations costs increased significantly since 2017, with a maximum growth rate of more than 40%. See Fig. 2 and Table 2.

**Table 2:** Trend in total hospital costs for strabismus patients, 2014–2019

Costs	2014	2015	2016	2017	2018	2019	P*
^−^X ± SD	4111 ± 1049	4492 ± 1049	4835 ± 1080	5884 ± 1467	6734 ± 1408	7023 ± 1380	
β ± SE	0 (ref.)	437.2 ± 68.1	762.5 ± 66.2	1802.3 ± 68.2	2560.3 ± 72.7	2846.2 ± 83.1	613.2 ± 13.6

^*−*^*X* mean; *SD* standard deviation; *β* regression coefficient; *SE* standard error

P*: significance of the change trends across years

### The composition ratio of hospitalization expenses for strabismus patients

**Fig. 3:**
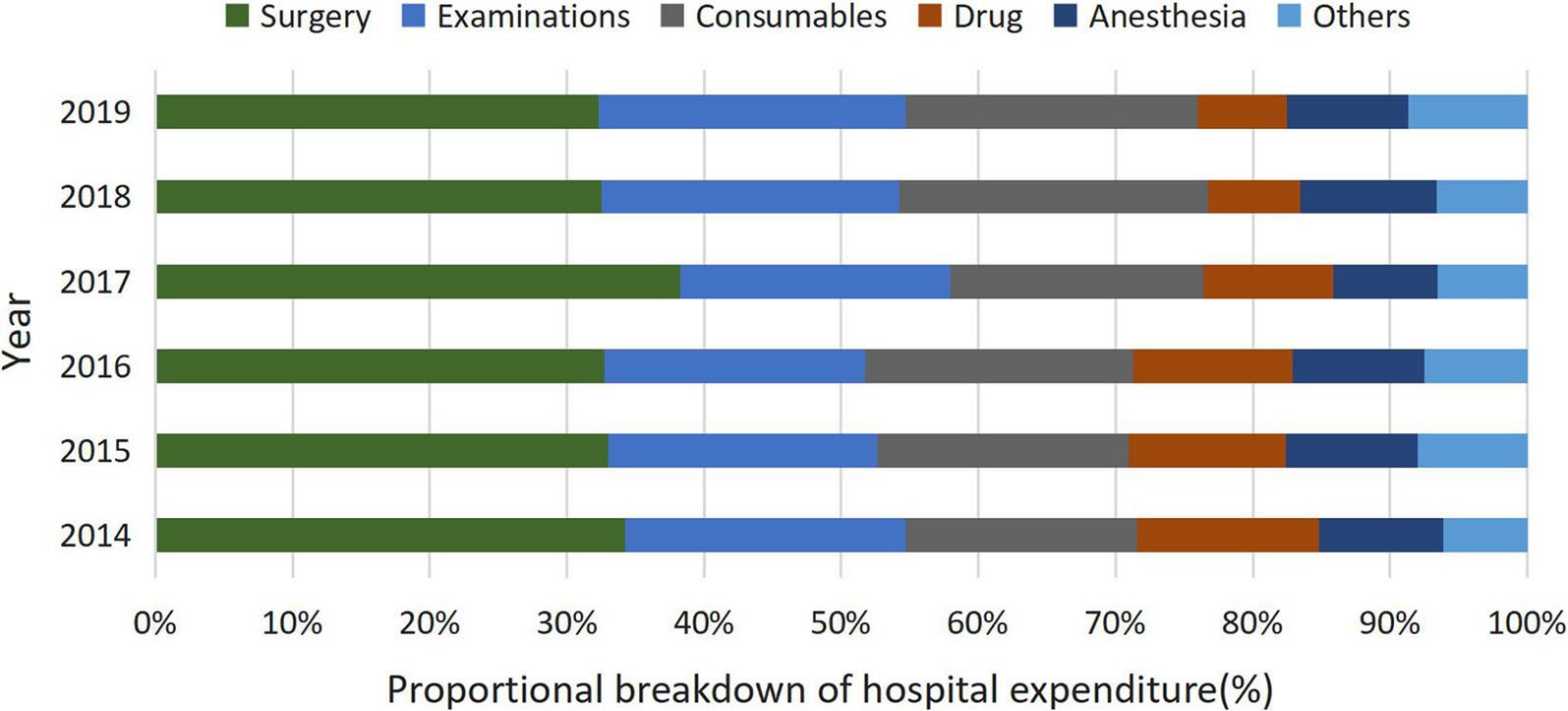
Changes in the composition of strabismus hospitalization costs in different years

The percentage of surgical treatment costs was the highest approximately 42.0%, followed by examinations and consumables costs, as shown in the Table 3. The percentage of surgery and anaesthesia costs decreased slightly over time. The proportion of examinations and consumables costs increased almost year by year. The proportion of drug costs was on a decreasing trend (Fig. 3). The cost of surgical treatment had the highest correlation with total costs, followed by the cost of drug and medical consumables (Table 3).

**Table 3:** Correlation and composition of various inpatient costs to total costs

Costs	Percentage $\overline{X}$ ± *SD*	Spearman’s correlation coefficient^a^
General medical service	0.0504 ± 0.0441	− 0.01900
General treatment operation	0.0056 ± 0.0050	0.72421
Nursing	0.0078 ± 0.0072	0.49969
Other	0.0060 ± 0.0040	0.28483
Laboratory test	0.0940 ± 0.0291	0.04636
Clinical diagnosis project	0.0137 ± 0.0150	− 0.53679
Imaging	0.0896 ± 0.0309	0.11433
Drug	0.1010 ± 0.0467	0.74986
Consumables for treatment	0.0697 ± 0.0216	0.74092
Consumables for surgery	0.1170 ± 0.0295	0.74961
Surgical treatment	0.4195 ± 0.0659	0.86107
Anesthesia	0.0889 ± 0.0663	0.81633
Surgery	0.3306 ± 0.0859	0.52629

^a^Spearman’s correlation coefficient, adjusted for CPI

### The influencing factors of hospitalization costs in strabismus patients

#### Univariate analysis

Age, gender and anesthesia were significant difference with total costs. There was no statistically significant difference about length of stay and funding, as shown in Tables 4 and 5.

**Table 4:** The influence of measurement data on total costs of hospitalization

Variables	Regression coefficients	t	*P*
Age	− 59.465	− 29.219	0.000
Length of stay	− 1.400	− 0.065	0.949

**Table 5:** The influence of enumeration data on total costs of hospitalization

Variables	Groups	Number of participants	Median (Interquartile range)	Statistics	*P*
Gender	Male	1704	5287.00 (1997.47)	− 2.465	0.014
	Female	1892	5169.81 (2039.55)		
Funding	Medical insurance	433	5287.68 (1823.79)	4.659	0.097
	Self-fund	3004	213.78 (2014.26)		
	Others	159	5430.93 (2345.04)		
Anesthesia	Local anaesthesia	1543	3972.25 (1744.76)	− 34.390	0.000
	General anaesthesia	2053	5675.79 (2073.72)		

#### Multi-factor analysis

Age and the total cost show negative correlation, that is, the older the age, the lower the total cost. At the same time, we found a positive correlation between the total cost and general anesthesia. By the partial correlation analysis, anaesthesia costs were most closely associated with changes in total hospital costs. Therefore, anaesthesia techniques is the most important factor affecting the cost of strabismus hospitalization. The results of the multivariate analysis are shown in Table 6.

**Table 6:** Results of multiple regression analysis of factors influencing total hospital costs

Variables	Regression coefficient	t	*P*
Gender—female	− 31.216	− 0.785	0.432289
Anesthesia—general anaesthesia	1804.481	34.167	0.000
Age	− 7.678	− 3.470	0.000

## Discussion

Strabismus surgery has been shown to be cost-effective and “very cost-effective” [31, 32], with similar health benefits to cataracts [31]. This is the first comprehensive study to reflect the current situation of medical costs associated with strabismus surgery by analyzing the trend of strabismus surgery costs and its influencing factors.

In this study, the number of patients under the age of 12 still accounts for the largest proportion, about 48.4%. It is probably traceable to the increasing attention to children’s health, increasing consultation and screening rates of children as higher living standards in China [33]. Most children receive treatment in the early stages of the disease. Kvarnström et al. pointed out that the prevalence of strabismus was highest in 4 -year-old children, and then showed a downward trend [8]. This shows that strabismus is still a common disease in children [34]. In terms of funding, over 80% of the patients in this study paid fees themselves. All types of strabismus have not been included in the scope of medical insurance reimbursement except paralytic strabismus in China. As we know, the main purpose of strabismus treatment is to improve the binocular visual function and psychosocial function [35, 36]. Strabismus surgery not only is a cosmetic procedure, but also is a functional restoration procedure [29]. Therefore, it is recommended that strabismus should be brought into the scope of medical insurance in China. Medical insurance can supervise medical services, guide medical institutions to take the initiative to control costs and provide early treatment to more patients, especially those with a heavier financial burden [37]. The early diagnosis and treatment of strabismus are essential to restore visual function and reduce the risk of amblyopia [38].

This study found that the total cost of hospitalization for strabismus increased over time. The year-on-year growth rate of hospitalization expenses in 2017 and 2018 was significantly higher than those in 2015 and 2016. The most of the sub-costs kept increasing, especially the costs of consumables and examinations, which leading to a significant increase in the total hospitalization costs. The only sub-costs that have declined were the costs of drug (particularly Western medicine) and general medical service, which may be influenced by the zero-mark-up medicines policy that has been fully implemented in the hospital since 2017. In China, a series of policies about the zero-mark-up medicines is the part of the new health-care reform, including abolition of drug mark-ups in all public hospitals, reduction of the prices of medical consumables and examination, raising the prices of items that reflect the value of the technical services of medical staff, such as surgery, nursing, etc. [39]. The general goal of the reforms were the growth rate of medical expenses in public hospitals reduced to less than 10% [39]. The hospital fell short of its goal, from the data of the study. There are many possible reasons: (1) the relatively slow increase in the cost of medical items reflecting the professional value, and the rapid reduction for drug costs (surgery costs showed a negative increase in 2018, while drug costs fell at a maximum rate of 22.5%).Those led to excessive examinations, and the total costs did not decrease but increase [40, 41]. (2) The price and amount of consumables used are not clearly specified, which may lead to high consumables costs and frequent use of consumables [42]. (3) Strabismus is not included in the medical insurance coverage, so medical insurance policy cannot be used to restrain medical behavior.

The cost of surgical treatment showed a downward trend except for a slight increase in 2017. The proportion of examinations costs and consumables costs have increased year by year, which was unreasonable and resulted in the continuous increase of the total cost of hospitalization.

The study shows the main factors, which influence the total of hospitalization for strabismus, are consumables costs, type of anesthesia and age. Consumables costs had a greater impact on the change of hospitalization costs. Therefore, it is necessary to reduce consumables costs and examinations costs, and to increase the costs for medical services, such as nursing costs, treatment costs and operation costs, etc. [43]. In particular, it is necessary to increase the cost of surgery reasonably. Compared with cataract, strabismus surgery was performed by two doctors. So operation costs should be more higher [29], in order to reflect the labour value of the medical staff.

The different anaesthesia techniques affect the total cost of hospitalization and the proportion of each sub-costs. The cost of general anaesthesia is significantly higher than that of local anaesthesia. General anaesthesia is often used for strabismus surgery in some countries and regions [29]. However, we believe that topical anaesthesia combined with local infiltration should be used as far as possible when the patients can cooperate and tolerate the procedure. There are three possible explanations. The first, topical anaesthesia can avoid the risks of general anaesthesia and other local anaesthetic techniques [44], reduce the incidence of oculocentric reflexes [45], post-operative nausea and vomiting [46, 47], and recover faster for post-operative patients. In addition, with the cooperation of the patients during the operation, the strabismus surgery with one-stage adjustable sutures can bring better effect and long-term stability [48, 49]. At last, it can reduce the total cost of hospitalization for strabismus and the economic burden of patients.

The multivariate analysis showed that the younger the age, the higher the cost of hospitalization. Patients with strabismus at different ages may have an impact on the total cost of hospitalization in various ways. The proportion of patients (≤ 12 years old) under general anaesthesia is over 90%In addition, the costs of items, such as blood collection and nursing are charged at an additional 20–30% for children(≤ 6 years old).

The optimal timing of strabismus surgery is still controversial, but it is widely accepted that strabismus surgery should be performed before children’s visual maturity (before 8 years old) [50]. Although the younger the child is, the greater the cost of strabismus surgery, from the perspective of patients early surgery can improve visual function without adverse long-term effects.

Age and the mode of anesthesia interact to some extent and work together on the total hospital costs. For example, 12-year-old strabismus patients mostly use general anesthesia (> 90%), with the average hospitalization cost of 6343.4 CNY. In addition, the proportion of general anesthesia in patients aged 13–24 years was about 42.6%, with the average hospitalization cost of 5352.8 CNY.

In this study, the length of stay did not have a statistically significant effect on hospitalization costs. However, previous studies have shown that the length of stay of many diseases heavily influenced hospitalization costs [51–53]. This may be due to the fact that the majority of strabismus patients are children with no other concomitant illnesses which incur additional costs. In addition, post-operative examination cannot be required for strabismus during hospitalization and only items with lower fees were charged, such as consultation and nursing fees. Besides, the short duration of strabismus surgery and the absence of serious post-operative complications make it suitable for day ward management. The day ward shortens the length of stay, reduces the stress, anxiety and mental strain caused by prolonged hospitalization, and improves patient satisfaction. The shorter length of hospital stay also speeds up bed turnover, freeing up more beds for critically ill patients and greatly improving bed utilization efficiency [54].

## Limitations

In this study, we aimed to estimate direct medical costs of strabismus patients based on medical records, while indirect and intangible costs were not included. This may lead to an underestimation of the medical costs associated with strabismus and an inability to accurately assess the financial burden on patients with strabismus. In addition, although a large sample was collected in this study, it still needs to be further demonstrated by multi-center studies.

## Conclusion

To our knowledge, this is the first study to perform trend analysis on the economic evidence of surgery for persons with strabismus. The total costs of hospitalization for strabismus had been increasing year by year and the growth rate is relatively fast. The general medical service costs and drug costs showed a downward trend, but the costs of medical consumables and examinations showed an increasing trend. Surgical costs account for the largest proportion, followed by examinations costs and medical consumables costs. In this study, we found that the mode of anaesthesia was the most significant influencing factor and age was negatively associated with the costs of hospitalization. Surgical treatment expenses (included operation and anesthesia expenses) had the greatest correlation with changes in total hospitalization expenses, followed by medicine expenses and medical consumables expenses. Reducing the costs of surgery, medical consumables and examinations are the means that can reduce the economic burden of strabismus patients. So, more attention should be paid to how strabismus can be included in Chinese Medical Insurance System.

## Data Availability

All data are available on reasonable request. The data described in this article can be freely and openly accessed at Science Data Back: https://www.scidb.cn/s/rMrmUr.
